# Four Decades of Obesity Trends among Non-Hispanic Whites and Blacks in the United States: Analyzing the Influences of Educational Inequalities in Obesity and Population Improvements in Education

**DOI:** 10.1371/journal.pone.0167193

**Published:** 2016-11-28

**Authors:** Yan Yu

**Affiliations:** 1 Health Research Institute, University of Canberra, Bruce, ACT, Australia; 2 School of Demography, Australian National University, Acton, ACT, Australia; Hunter College, UNITED STATES

## Abstract

Both obesity (body mass index ≥ 30) and educational attainment have increased dramatically in the United States since the 1970s. This study analyzed the influences of educational inequalities in obesity and population improvements in education on national obesity trends between 1970 and 2010. For non-Hispanic white and black males and females aged 25–74 years, educational differences in the probability of being obese were estimated from the 1971–2012 National Health and Nutrition Examination Surveys, and population distributions of age and educational groups, from the 1970 Census and 2010 American Community Survey. In the total population, obesity increased from 15.7% to 38.8%, and there were increases in the greater obese probabilities of non-college graduates relative to four-year college graduates. The increase in obesity would have been lower by 10% (2.2 percentage points) if educational inequalities in obesity had stayed at their 1970 values and lower by one third (7.9 points) if obesity inequalities had been eliminated. Obesity inequalities were larger for females than males and for whites than blacks, and obesity did not differ by education among black males. As a result, the impact of obesity inequalities on the obesity trend was largest among white females (a 47% reduction in the obesity increase if obesity inequalities had been eliminated), and virtually zero among black males. On the other hand, without educational improvements, the obesity increase would have been 9% more in the total population, 23% more among white females and not different in the other three subpopulations. Results indicate that obesity inequalities made sizable contributions to the obesity trends, and the obesity reductions associated with educational improvements were more limited.

## Introduction

Both obesity and educational attainment have increased dramatically in the last 40 years. In the United States, the prevalence of obesity (body mass index or BMI 30 and greater) increased from 15% in the 1970s to over one third in 2012[1, 2], whereas the proportion without a high school degree decreased from 45% to less than 10% (author’s calculation based on the 1970 Census and 2010 American Community Survey). Obesity is associated with a host of metabolic complications and chronic conditions (e.g., diabetes, cardiovascular disease and cancer), and elevates disability and mortality[3, 4], whereas post-secondary education is conducive to favorable health outcomes[5, 6]. Despite the obesity epidemic, the most educated people are least likely to be obese[7]. Early trend analyses found that the most educated groups have experienced the greatest increases in obesity, and the negative education-obesity association has weakened over time[8–11]; however, recent studies found that although obesity has increased for all educational groups, the education-obesity linkage has remained unchanged or become stronger[12–15]. It was further argued that the greatest obesity increases have occurred to the medium educational groups (those who have attended college but not completed a four-year college education), and lumping them together with the four-year college group has led to the artefact of a weaker education-obesity association[12].

The persistent or increased educational inequalities in obesity suggest that health programs targeting the less educated groups could reduce national obesity levels. However, there was doubt about this approach[16]. Empirically, it is poorly understood how obesity inequalities and levels are related. One study found that had all educational groups experienced the same proportional increase in obesity between 1984 and 1994, the 1994 obesity level would have been 2.6 percentage points lower in the United States[10]. This analysis, however, was biased by grouping together the two heterogeneous college groups (with and without a four-year college education). In addition to inequalities in individuals’ obesity risk, educational attainment affects national obesity levels through shifts in the population distribution. Presumably, if the negative education-obesity association prevails throughout the educational spectrum, population improvements in education (e.g., by shifting more people to higher educational levels) should slow down the obesity epidemic, even without altering educational inequalities in the obesity risk. On the other hand, such educational improvements may not reduce national obesity levels if the educational category that has become more numerous in the population has also become more likely to be obese, relative to others. No prior research has examined the impact of population educational improvements on national obesity levels and trends.

This paper analyzed the influences of educational attainment on national obesity trends from the perspectives of 1) educational inequalities in obesity and 2) population distributions of educational categories. Educational differences in the probability of being obese were estimated from the 1971–2012 National Health and Nutrition Examination Surveys (NHANES), and population distributions of age and educational attainment from the 1970 Census and 2010 American Community Survey for US non-Hispanic white and black males and females. Hypothetical scenarios were constructed to quantify the respective impacts on obesity trends of obesity inequalities and population distributions of education. The obesity epidemic has shown no signs of reversal in the United States or elsewhere[17]. Educational differences in obesity have been documented for various populations, and improvements in the level and quality of educational attainment are increasingly regarded as a way to improve population health[18, 19]. An improved understanding of the different aspects of education influences on obesity is needed to understand obesity and health trends and inform health interventions.

## Materials and Methods

The NHANES series consists of independent cross-sectional multistage stratified probability samples that represent the U.S. non-institutionalized population. Publicly available data for 10 samples were analyzed: NHANES I collected in 1971–74, NHANES II in 1976–80, NHANES III in 1988–94 and seven two-year cycles from the continuous NHANES in 1999–2012[20–23]. The analysis did not include the 1959–62 National Health Examination Survey because its education variable combined three and four years of college into one category and did not allow for distinguishing the four-year college group.

Body weight and height were measured by health professionals during the NHANES physical exam. They were used to define BMI as weight in kilograms divided by the square of height in meters, and obesity as BMI ≥ 30 kg/m^2^[24]. Educational attainment was classified into four levels: less than high school (completed grade less than 12), high school (grade 12, high school graduation, General Educational Development[GED] or equivalent), some college (13–15 years of school or associate’s degree), and at least four-year college (at least 16 years of school or bachelor degree, as reference). The high school degree category included both high school graduates and recipients of the GED high school equivalency credentials because the two subgroups could not be separated in the NHANES data. Survey year was available for NHANES I and II, and approximated as the midpoint of survey phase for NHANES III (1990 for Phase I and 1993 for Phase II) and the second year of each two-year cycle in the continuous NHANES.

The analysis was restricted to non-Hispanic whites and blacks aged 25–74 years. Information on national origin was used to identify Hispanics (or rather, non-Hispanics) in the first two NHANES, which did not collect information on ethnicity. The NHANES I and II samples were too small for Hispanics and for other racial/ethnic groups to be included in the analysis. Of the 48,275 respondents meeting the sample selection criteria, 728 females were pregnant, 343 cases missing for anthropometric measures and 285 cases missing for education. After their deletions, the final analysis sample had 46,919 cases (18,245 white females, 16,018 white males, 6,842 black females and 5,814 black males).

The linear probability model was used to examine educational differences in the obese probability, as done previously[13]. For the combined sample of whites and blacks, we estimated a model that included sex, race/ethnicity, educational attainment, linear and quadratic terms for age and survey year, and interactions between education and survey year (linear term). Model specification details were shown in S1 Text. Under this model, the obese probability varies non-linearly with age and time, and educational differences in obesity change over time. Preliminary analyses did not find evidence of non-linear time trends in the obesity differences by educational groups.

As educational differences in obesity may vary between the sexes and racial/ethnic groups, the linear probability model was further fitted to the four sex-race subsamples, but excluding the sex and race variables, the quadratic terms for survey year for the female samples, and the education-survey year interactions for black males. The last two sets of terms were excluded because they were found statistically non-significant at any conventional levels in preliminary analyses. Under these exclusions, obesity increased linearly over time for females, and educational differences in obesity did not change over time for black males. All analysis used sample weights, and survey design effects (clustering and stratification) were accounted for by the Generalized Estimating Equations framework[25]. The design variables were recoded to have unique values across surveys; original NHANES weights were rescaled to sample size within each survey.

National obesity level at any time was as a function of 1) the proportions of people in the subpopulations (i.e., population distributions) and 2) the obese probabilities within the subpopulations. Here, the subpopulations were defined with respect to sex, race/ethnicity, age and educational attainment. A mathematical expression of this function was shown in S2 Text. The NHANES estimates were used for the obese probabilities, and data from the 1970 Census and 2010 American Community Survey[26], for population distributions of age, sex, race/ethnicity and educational attainment.

To analyze the impact on the obesity level of population improvements in education, we asked the hypothetical question of what the 2010 national obesity level would have been if the population education distribution had been maintained at its 1970 values (Scenario 1). Comparing the hypothetical against actual values would quantify the influence of the changing population education distribution on national obesity level. To reveal the impacts of obesity inequalities, hypothetical 2010 national obesity levels were estimated by manipulating the obese probabilities under three scenarios that used the 1970 values of obese probabilities within educational groups (Scenario 2) or 1970 values of educational differences in obesity (Scenario 3), or eliminated educational differences in obesity (Scenario 4). Scenario 3 differed from Scenario 2 in allowing the obese probabilities within educational groups to change (here, increase) from 1970 to 2010. Both Scenarios 3 and 4 used the 2010 actual obese probabilities for the four-year college group, and allowed the obese probabilities within all educational groups to increase over time.

To compare the 1970 and 2010 obesity levels and analyze obesity trends, an age-standardized 1970 obesity level was also calculated, using the 2010 age distribution as the standard. The standardized and hypothetical obesity levels were estimated for the combined population of whites and blacks and separately for the four sex-race subpopulations. For the combined population, strictly speaking, obesity trends were further affected by changes in the population distributions of sex and race. However, as shown below, these changes were too small to seriously bias obesity trends.

## Results

Table 1 presents the sample size and obesity level by educational attainment across the 10 NHANES surveys for each of the four sex-race-specific subsamples. Obesity increased over time for all groups, and the less educated were more likely to be obese than the four-year college group. The sample size was smaller for the black than white samples, and in NHANES I, only 38 black females and 19 black males were in the four-year college group.

**Table 1:** Sample size (unweighted *N*) and obesity risk (% body mass index ≥ 30 kg/m^2^), by educational attainment, U.S. non-Hispanic whites and blacks, aged 25–74.

	NHANES I 1971–1974		NHANES II 1976–1980		NHANES III 1988–1994		Continuous NHANES													
							1999–2000		2001–2002		2003–2004		2005–2006		2007–2008		2009–2010		2011–2012	
	N	%	N	%	N	%	N	%	N	%	N	%	N	%	N	%	N	%	N	%
**White female**																				
Total	5191	16.7	4485	16.5	2651	24.4	657	32.6	856	33.1	836	32.1	775	35.9	979	33.8	1108	34.9	707	34.8
<hs^1^	2117	23.6	1668	23.5	584	31.1	104	39.2	111	45.7	105	38.3	84	40.6	166	48.1	187	40.5	89	41.2
hs^2^	2018	15.1	1732	15.1	1060	27.7	199	39.0	226	33.3	248	33.9	192	41.3	264	36.9	264	41.1	130	45.4
somcol^3^	556	9.2	589	13.1	506	22.9	185	33.6	270	36.1	273	33.9	254	41.2	306	37.0	334	38.6	252	36.1
≥4-yr col^4^	500	7.7	496	8.6	501	14.6	169	21.2	249	25.0	210	25.6	245	25.3	243	22.2	323	25.6	236	27.9
**White male**																				
Total	3494	12.5	4078	12.7	2327	21.4	710	27.7	898	30.5	865	32.4	843	34.4	1007	34.8	1058	38.1	738	35.1
<hs^1^	1671	13.7	1545	14.3	560	27.3	127	29.9	129	28.7	122	27.5	104	37.1	196	39.3	162	34.4	121	36.4
hs^2^	987	15.1	1301	15.2	738	22.7	192	32.6	229	32.4	244	34.0	238	40.6	281	33.3	265	39.8	156	37.8
somcol^3^	345	8.4	508	12.8	418	20.9	177	28.4	229	30.6	270	37.3	246	35.8	267	39.9	318	48.0	230	40.5
≥4-yr col^4^	491	8.1	724	6.9	611	16.5	214	21.5	311	29.5	229	27.2	255	26.8	263	29.2	313	28.8	231	29.0
**Black female**																				
Total	1194	31.4	610	32.2	2028	39.2	355	50.7	362	49.7	354	56.6	433	53.5	497	50.6	437	60.2	572	61.0
<hs^1^	816	38.2	382	40.3	679	45.5	134	45.8	120	52.8	103	56.9	104	56.0	142	52.4	106	58.9	99	66.0
hs^2^	268	21.9	148	25.5	776	39.2	86	55.1	83	44.8	96	58.1	90	50.6	112	52.7	109	54.5	143	57.7
somcol^3^	72	25.5	43	18.2	357	36.0	94	56.2	109	55.8	105	58.7	159	57.5	152	54.0	160	64.2	201	67.9
≥4-yr col^4^	38	12.4	37	21.3	216	29.2	41	43.6	50	38.2	50	48.9	80	45.6	91	40.4	62	62.6	129	51.0
**Black male**																				
Total	695	18.2	508	16.0	1698	21.8	316	28.9	363	27.4	348	35.5	422	37.5	475	38.6	454	41.5	535	37.9
<hs^1^	527	15.0	331	16.6	664	21.4	139	34.0	131	21.4	100	28.0	115	37.0	146	26.0	106	37.5	128	34.9
hs^2^	106	22.8	100	19.0	580	21.1	63	28.3	91	27.0	86	34.9	118	40.1	123	46.5	143	41.9	155	37.0
somcol^3^	43	31.9	47	9.7	299	22.5	74	23.7	87	35.2	109	39.2	123	35.6	134	38.8	137	46.5	153	36.5
≥4-yr col^4^	19	13.1	30	13.6	155	24.3	40	23.8	54	27.0	53	42.4	66	37.2	72	45.1	68	37.1	99	44.9

^1^less than high school

^2^high school degree

^3^some college

^4^at least four-year college

Estimates from the linear probability models are shown in Table 2. Obesity increased over time and age, and was higher for blacks than whites and for females than males. For the combined sample of whites and blacks, compared with the four-year college group, obesity was higher for the two high school groups (with and without a degree), but not for the some college group in 1970. The interactions between education and survey year were positive, and for the high school degree and some college groups, the interactions were statistically significant, indicating that over the 40 years, the obese probabilities became increasingly higher for the two medium educational groups, relative to those with a four-year college.

**Table 2:** Estimates (standard errors) from linear probability models of obesity (body mass index ≥ 30 kg/m^2^), U.S. non-Hispanic whites and blacks, aged 25–74 years, 1971–2012 NHANES.

Parameters		Whites & blacks	White female	White male	Black female	Black male
Intercept		0.1216*	0.0960*	0.0945*	0.1368*	0.2175*
		(0.0094)	(0.0132)	(0.0137)	(0.0382)	(0.0304)
Blacks (vs. whites)		0.0942*	—	—	—	—
		(0.0071)				
Male (vs. female)		-0.0287*	—	—	—	—
		(0.0046)				
Age in years (*A*)						
	*A* (centered at 50)	0.0013*	0.0018*	0.0009*	0.0016*	0.0007
		(0.0002)	(0.0002)	(0.0002)	(0.0006)	(0.0005)
	*A*^*2*^ (centered at 50)	-0.0001*	-0.0001*	-0.0001*	-0.0001*	-0.0000
		(0.0000)	(0.0000)	(0.0000)	(0.0000)	(0.0000)
Year of survey (*T*)						
	*T* (centered at 1970)	0.0020*	0.0045*	0.0019	0.0099*	-0.0025
		(0.0010)	(0.0005)	(0.0013)	(0.0012)	(0.0021)
	*T*^*2*^ (centered at 1970)	0.0001*	—	0.0001*	—	0.0002*
		(0.0000)		(0.0000)		(0.0000)
Education (vs. at least 4-year college)						
	< high school (hs)	0.0826*	0.1261*	0.0504*	0.2543*	-0.0432
		(0.0110)	(0.0191)	(0.0144)	(0.0395)	(0.0228)
	hs	0.0540*	0.0539*	0.0697*	0.1143*	-0.0050
		(0.0108)	(0.0152)	(0.0145)	(0.0399)	(0.0201)
	Some college (somcol)	0.0130	0.0113	0.0199	0.0664	0.0016
		(0.0135)	(0.0178)	(0.0183)	(0.0603)	(0.0230)
Interaction (X) terms						
	*T* X <hs	0.0007	0.0010	0.0007	-0.0048*	—
		(0.0005)	(0.0009)	(0.0008)	(0.0014)	
	*T* X hs	0.0014*	0.0022*	0.0003	-0.0012	—
		(0.0005)	(0.0007)	(0.0007)	(0.0015)	
	*T* X somcol	0.0027*	0.0030*	0.0023*	0.0015	—
		(0.0005)	(0.0007)	(0.0008)	(0.0020)	

*p<0.05

Across the four subsamples, obesity was higher for the two high school groups among whites and black females in 1970, and became increasingly higher over time for the high school degree and some college groups among white females and for the some college group among white males, relative to the four-year college group. Obesity also increased for black females with some college relative to those with a four-year college, but statistical uncertainty was high, and the trend estimate was statistically non-significant. Obesity did not differ by education among black males over the study period. In general, statistical uncertainty was two to three times higher for the black than white estimates, reflecting sample size differences across the subsamples (Table 1).

**Fig 1:**
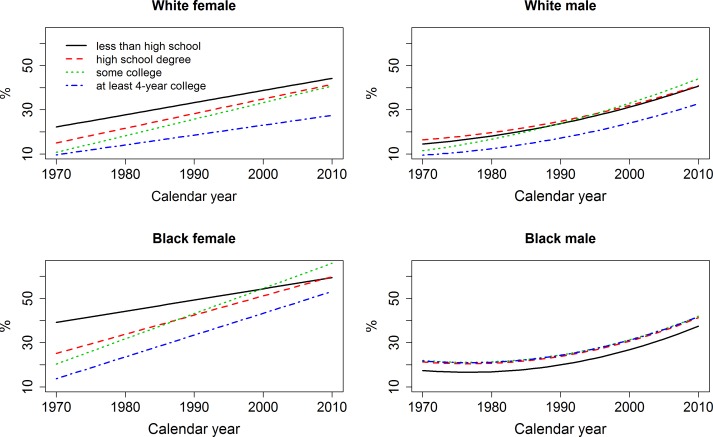
Trends in obesity (body mass index ≥ 30 kg/m^2^), by educational attainment, U.S. non-Hispanic blacks and whites, aged 50 years, 1971–2012 NHANES

Fig 1 shows the estimated obese probabilities by educational attainment for the four subsamples. Among whites and black females, the increases in obesity between 1970 and 2010 were larger for the medium educational groups than the least or most educated; and the obese probabilities diverged between college graduates and the three groups of non-college graduates, and converged among the latter three. In 2010, obesity was highest for high school dropouts among white females and for the some college group among white males and black females, but the differences among the non-college graduates were statistically non-significant (not shown).

The 1970 and 2010 population distributions of sex and race/ethnicity were shown in S1 Table and the distributions of age and educational attainment for each of the four sex-race subpopulations, in S2 Table. In the combined population, the proportions of blacks increased between 1970 and 2010, but the increase was less than 3 percentage points (from 5.4% to 8.0% for black females and 4.5% to 6.9% for black males). All four subpopulations became somewhat older: The percentages of those under 45 were 1–3 points lower in 2010 than in 1970. Educational improvement was substantial. In 1970, only 11–29% of white and black males and females had attended college. The corresponding figures reached 46–66% in 2010. Among the college attendants in 2010, about half of the whites and two-thirds of the blacks did not have a four-year college.

**Fig 2:**
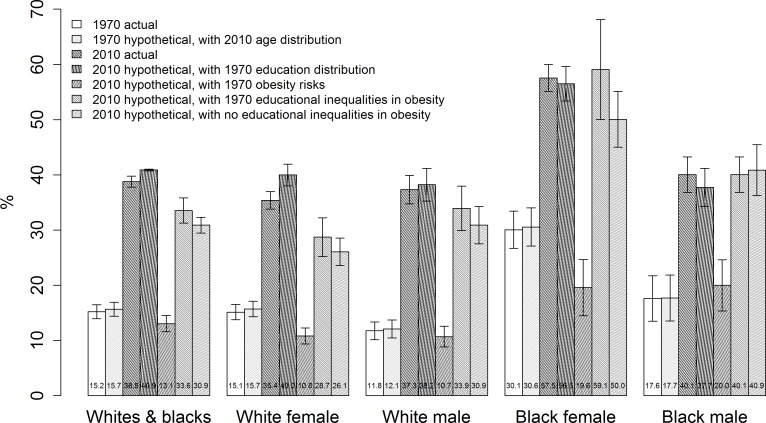
Actual vs. hypothetical obesity level (body mass index ≥ 30 kg/m^2^), 1970 and 2010, US non-Hispanic whites and blacks, aged 25–74, 1971–2012 NHANES Notes: Numbers at the bottom are the central values of obesity levels and error bars, 95% CIs; the last two hypothetical scenarios used the actual values for the 2010 obesity risk of four-year college graduates.

Fig 2 shows the actual and hypothetical obesity levels for the five populations in 1970 and 2010. Numerical estimates were also shown in tabular form in S3 Table. Over the 40 years, obesity level increased by two folds or more. The 1970 actual obesity levels were < 1 percentage point lower than the values standardized by the 2010 age distribution, indicating that population aging played a trivial role in the obesity increases. A comparison of the 1970 age-standardized and 2010 actual obesity levels showed that obesity increased by 23.1 percentage points in the combined population, 19.7 points among white females, 25.2 points among white males, 26.9 points among black females and 22.4 points among black males.

What was the role of educational improvements in the obesity trends? If there had been no educational improvements and the 1970 education distributions had stayed unchanged over the 40-year period (Scenario 1), the 2010 obesity level would have been 2.1 percentage points higher in the combined population and 4.6 percentage points higher among white females, and not different in the other three subpopulations. Thus, population improvements in educational attainment were associated with a 9% reduction of the obesity increase in the combined population, and across the four subpopulations, a 23.4% reduction among white females only.

On the other hand, if the 1970 obesity risks within each educational group had prevailed in 2010 (Scenario 2), the 2010 national obesity level would have been 20–38 percentage points lower across the five populations. Given the secular rise in obesity, it may be more reasonable to ask what the 2010 obesity level would have been if obesity had increased for all educational groups, but the increase of the less educated groups had been no larger than that of the four-year college group such that educational differences in obesity had remained at their 1970 values (Scenario 3) or had been eliminated in 2010 (Scenario 4). Under the scenario of no changes in the obesity differences across educational groups, the 2010 obesity level would have been 2.2 percentage points lower in the combined population, 6.7 points lower among white females and not different in the other three subpopulations. Under the scenario of no obesity differences, the obesity reductions would reach 7.9 points in the combined population, 9.3 points among white females, 6.4 points among white males and 7.5 points among black females. These reductions took up sizable portions of the actual increases in obesity: 10–34% (Scenario 3) and 25–47% (Scenario 4).

## Discussion

Obesity increased from 15.7% in 1970 to 38.8% in 2010 among non-Hispanic whites and blacks in the United States. Over the 40 years, the greater obesity risks of non-college graduates relative to four-year college graduates increased, making a positive contribution to the obesity trends. The increase in obesity would have been reduced by 10% (2.2 out of the total increase of 23.1 percentage points) if educational inequalities in obesity had stayed constant at their 1970 values and reduced by one third (7.9 out of 23.1 percentage points) if obesity inequalities had been eliminated. Educational inequalities in obesity were generally larger for females than males and for whites than blacks, and obesity did not differ by educational attainment among black males. As a result, the impact of obesity inequalities on national obesity level was largest among white females (as large as a 47% reduction of the increase in obesity level if obesity inequalities were eliminated), and virtually zero among black males. The estimates for the two black subsamples were less precise because of smaller samples.

To our knowledge, this was one of the first studies to quantify the impact of increasing obesity inequalities on national obesity trends. The increasing obesity inequalities were primarily driven by the some college group, who did not differ from those with at least a four-year college in the 1970s, but had the largest obesity increase over the 40 years and had an obesity risk in 2010 that was as high as those of high school dropouts and high school degree holders. The greater obesity increase of the some college group was neglected in early analyses that lumped the two college groups into one category[8–11], but have been recognized in recent analyses[12, 15]. However, the implications of the widening obesity gap between college and non-college graduates for national obesity trends had gone largely unnoticed or hidden. As no population subgroups had been immune from the obesity increase, even analyses of inequalities placed the emphasis on the common aspect of the epidemic and regarded the greater obesity increases of subgroups as exceptions[13]. There was the opinion that the reduction of obesity inequalities may not be useful to control the obesity epidemic at all[16]. The scenarios constructed here allowed obesity to increase over time for all four educational groups, but the increases were smaller for non-college than college graduates. Estimates suggest sizable impacts of obesity inequalities on obesity trends, and the impacts were larger for subpopulations with larger obesity inequalities.

Educational attainment is associated with adult risk factors for obesity. The increasing obesity inequalities favoring the four-year college group are consistent with the increasing advantages associated with college graduates in a variety of adult outcomes such as wages and earnings[27, 28], marriage and divorce[29, 30] and health and mortality[31, 32]. Adult lifestyle factors such as time spent in exercising and watching TV and energy intake were associated with educational attainment[33]. The education-obesity association could also have origins that are cumulated over the life course. Parental education, for example, may affect not only the offspring’s college entrance and graduation[34, 35] but also dietary intake and physical activity[36, 37]. Long-term interrelated processes could drive the trajectories of both educational attainment and obesity.

Population improvements in educational attainment per se, however, may play a rather limited role in the obesity trends. If there had been no educational improvements, the 2010 obesity level would have been 2.2 percentage points higher in the combined population, and across the four subpopulations, 4.6 points higher among white females only. Under the post-World War II expansion of the education system, more people have accomplished post-secondary education; however, the some college group has become more likely to be obese, relative to other educational groups. Thus, the potential reductions in obesity were not realized. These results suggest that mere shifts in the education distribution may not be effective in containing the obesity epidemic.

There are several study limitations to consider. Obesity was defined in terms of one cut-off point based on body mass index. Previous studies analyzing other measures (e.g., severe obesity, mean BMI, BMI percentiles and waist circumference) found that trends in the educational differences are in general similar across measures[13, 14], but among men, the relationship between education and the 15th percentile of the BMI distribution is positive[13]. Further research should examine the macro impact of educational inequalities in other body build measures. The NHANES has the advantage of a consistent study design and physically measured anthropometric information, and is the gold standard for obesity research. However, the NHANES samples are somewhat small, and its education variable did not distinguish GED recipients from high school graduates, which could be a source of heterogeneity[38] that should be investigated with appropriate data. The analysis considered obesity inequalities with respect to education, but obesity is determined by complex multifactorial processes at various levels that are shaped by the social and physical environments[39]. The variations in the current findings across the sex-race subpopulations are suggestive of the role played by the larger context. The individual characteristics (e.g., income) and environmental features (e.g. physical activity and food environments) may or may not correlate with education, but are beyond the scope of the current analysis.

To conclude, obesity inequalities have shown no sign of weakening, but increased between college and non-college graduates, making sizable contributions to the obesity trends among non-Hispanic whites and blacks in the United States over the last 40 years. Population shifts in educational attainment, however, have played a more limited role in the obesity trends. Improvements in life styles and living conditions targeting the less educated groups could reduce both obesity inequalities and obesity levels.

## Supporting Information

S1 Text The linear probability model (DOCX)

S2 Text A mathematical expression of obesity level (DOCX)

S1 Table Population distributions of sex and race/ethnicity, US non-Hispanic whites and blacks (DOC)

S2 Table Population distributions of age and educational categories (%), US non-Hispanic whites and blacks (DOC)

S3 Table Actual vs. hypothetical obesity levels (95% CI), 1970 and 2010, US non-Hispanic whites and blacks, aged 25–74 (DOC)
