# Supplementary material for: Four Decades of Obesity Trends among Non-Hispanic Whites and Blacks in the United States: Analyzing the Influences of Educational Inequalities in Obesity and Population Improvements in Education
Source: PLoS One. 2016 Nov 28;11(11):e0167193. doi: 10.1371/journal.pone.0167193 (PMC5125692; doi:10.1371/journal.pone.0167193)
Supplement: S1 Text — (DOCX) [file pone.0167193.s001.docx]

S1 Text. The linear probability model

Linear probability models were estimated to examine educational (*E*) differences in the obese probability (*P*). For the combined sample of whites and blacks, the model was written as:

$P=\beta_{0}+\beta_{1}\cdot a+\beta_{2}\cdot a^{2}+\beta_{3}\cdot T+\beta_{4}\cdot T^{2}+\beta_{5}\cdot E+\beta_{6}\cdot E\times T+\beta_{7}\cdot F+\beta_{8}\cdot B,$ (1)

where *a* denotes age in years (centered at 50), *T* year of survey (centered at 1970), *F* females, *B* blacks, and “×” interaction between two variables. The coefficients denote vectors when the variables are of more than two levels. The coefficients of the linear probability model can be directly interpreted as probabilities or differences in probabilities. Under Equation (1), $\beta_{5}$ estimates educational differences in obesity in 1970, and $\beta_{6}$ changes over time in these differences. The two quadratic terms accommodate non-linear obesity trends with age and time.
