# Supplementary material for: Four Decades of Obesity Trends among Non-Hispanic Whites and Blacks in the United States: Analyzing the Influences of Educational Inequalities in Obesity and Population Improvements in Education
Source: PLoS One. 2016 Nov 28;11(11):e0167193. doi: 10.1371/journal.pone.0167193 (PMC5125692; doi:10.1371/journal.pone.0167193)
Supplement: S2 Text — (DOCX) [file pone.0167193.s002.docx]

S2 Text. A mathematical expression of obesity level

For any population, the obesity level at any time *P(O)* is a function of the proportions of people in subpopulations (population distributions) and the obese probabilities within the subpopulations. In this paper, the subpopulations were defined with respect to sex and race/ethnicity, age and educational attainment. The corresponding proportions were denoted as *P(S)* (the population distribution of sex and race), *P_s_(a)* (the age distribution within sex and race) and *P_sa_(E)* (the education distribution within sex, race and age groups). If we denote the obesity risk within subpopulations as *P_saE_(O)*, the obesity level in the combined population can be expressed as:

$$P(O)=\sum_{s} P(s)\sum_{a} P_{s}(a)\sum_{E} P_{sa}(E)\cdot P_{saE}(O).$$

All the above quantities pertain to either the year 1970 or 2010.

The above equation was also used to separately estimate the obesity levels of white females, white males, black females and black males, after taking out the first distributional component of *P(s)*. Note that all these equations are mathematical identities, not statistical models.
