# Supplementary material for: Four Decades of Obesity Trends among Non-Hispanic Whites and Blacks in the United States: Analyzing the Influences of Educational Inequalities in Obesity and Population Improvements in Education
Source: PLoS One. 2016 Nov 28;11(11):e0167193. doi: 10.1371/journal.pone.0167193 (PMC5125692; doi:10.1371/journal.pone.0167193)
Supplement: S1 Table — (DOC) [file pone.0167193.s003.doc]

**S1 Table. Population distributions of sex and race/ethnicity, US non-Hispanic whites and blacks, aged 25-74, 1970 Census and 2010 American Community Survey**

| Year | White female | White male | Black female | Black male | Total |
| --- | --- | --- | --- | --- | --- |
| 1970 | 47.0 | 43.1 | 5.4 | 4.5 | 100.0 |
| 2010 | 43.1 | 42.1 | 8.0 | 6.9 | 100.0 |
