# Supplementary material for: Four Decades of Obesity Trends among Non-Hispanic Whites and Blacks in the United States: Analyzing the Influences of Educational Inequalities in Obesity and Population Improvements in Education
Source: PLoS One. 2016 Nov 28;11(11):e0167193. doi: 10.1371/journal.pone.0167193 (PMC5125692; doi:10.1371/journal.pone.0167193)
Supplement: S2 Table — (DOC) [file pone.0167193.s004.doc]

**S2 Table. Population distributions of age and educational categories (%), US non-Hispanic whites and blacks, 1970 Census and 2010 American Community Survey**

|  | Year 1970^1^ | | | | | |  | Year 2010^1^ | | | | | |
| --- | --- | --- | --- | --- | --- | --- | --- | --- | --- | --- | --- | --- | --- |
| Age groups | Of total |  | Educational groups | | | |  | Of total |  | Educational groups | | | |
|  |  |  | <hs^2^ | hs^3^ | sc^4^ | 4ycol^5^ |  |  |  | <hs^2^ | hs^3^ | sc^4^ | 4ycol^5^ |
| White female | |  |  |  |  |  |  |  |  |  |  |  |  |
| 25-29 | 12.4 |  | 22.4 | 45.2 | 17.5 | 14.9 |  | 9.6 |  | 4.7 | 19.7 | 33.8 | 41.7 |
| 30-34 | 10.5 |  | 26.9 | 46.3 | 15.0 | 11.7 |  | 9.1 |  | 4.5 | 19.9 | 33.6 | 42.0 |
| 35-39 | 10.3 |  | 30.8 | 45.8 | 13.8 | 9.7 |  | 9.4 |  | 4.4 | 21.4 | 34.2 | 40.0 |
| 40-44 | 11.2 |  | 35.3 | 42.8 | 12.9 | 9.0 |  | 10.5 |  | 4.6 | 25.0 | 33.2 | 37.2 |
| 45-49 | 11.8 |  | 37.4 | 42.7 | 12.6 | 7.4 |  | 12.2 |  | 5.0 | 28.8 | 34.6 | 31.6 |
| 50-54 | 11.0 |  | 44.5 | 37.3 | 10.9 | 7.3 |  | 12.7 |  | 5.2 | 30.5 | 34.1 | 30.2 |
| 55-59 | 10.0 |  | 52.6 | 29.6 | 10.5 | 7.2 |  | 11.7 |  | 5.2 | 30.4 | 33.0 | 31.4 |
| 60-64 | 8.8 |  | 58.5 | 23.4 | 10.6 | 7.5 |  | 10.6 |  | 6.6 | 33.0 | 31.2 | 29.1 |
| 65-69 | 7.6 |  | 64.9 | 19.2 | 9.8 | 6.1 |  | 8.1 |  | 9.6 | 38.7 | 28.6 | 23.2 |
| 70-74 | 6.2 |  | 69.5 | 16.6 | 8.9 | 5.0 |  | 6.2 |  | 13.1 | 42.4 | 25.6 | 18.9 |
| Total | 100.0 |  | 41.8 | 36.7 | 12.6 | 8.9 |  | 100.0 |  | 5.9 | 28.5 | 32.6 | 33.0 |
|  |  |  |  |  |  |  |  |  |  |  |  |  |  |
| White male | |  |  |  |  |  |  |  |  |  |  |  |  |
| 25-29 | 13.2 |  | 22.4 | 35.3 | 20.3 | 21.9 |  | 10.0 |  | 6.5 | 28.2 | 33.3 | 32.0 |
| 30-34 | 11.1 |  | 26.8 | 35.6 | 16.8 | 20.8 |  | 9.5 |  | 6.3 | 28.1 | 31.6 | 33.9 |
| 35-39 | 10.8 |  | 31.2 | 33.8 | 14.5 | 20.4 |  | 9.8 |  | 6.0 | 28.3 | 31.0 | 34.6 |
| 40-44 | 11.7 |  | 39.1 | 29.9 | 13.4 | 17.7 |  | 10.8 |  | 6.2 | 30.5 | 29.7 | 33.6 |
| 45-49 | 11.9 |  | 40.5 | 31.0 | 13.1 | 15.4 |  | 12.4 |  | 7.2 | 33.5 | 28.6 | 30.7 |
| 50-54 | 11.1 |  | 47.4 | 29.5 | 11.5 | 11.5 |  | 12.7 |  | 7.1 | 33.6 | 29.2 | 30.1 |
| 55-59 | 10.0 |  | 55.9 | 23.9 | 9.8 | 10.3 |  | 11.5 |  | 6.4 | 29.1 | 30.7 | 33.8 |
| 60-64 | 8.5 |  | 63.4 | 17.9 | 9.3 | 9.4 |  | 10.3 |  | 6.8 | 26.4 | 29.4 | 37.4 |
| 65-69 | 6.7 |  | 70.9 | 13.4 | 7.9 | 7.8 |  | 7.5 |  | 10.2 | 30.5 | 26.5 | 32.8 |
| 70-74 | 5.0 |  | 73.9 | 12.1 | 7.3 | 6.7 |  | 5.5 |  | 13.1 | 33.6 | 24.1 | 29.1 |
| Total | 100.0 |  | 43.4 | 28.1 | 13.2 | 15.3 |  | 100.0 |  | 7.2 | 30.2 | 29.7 | 32.9 |
|  |  |  |  |  |  |  |  |  |  |  |  |  |  |
| Black female | |  |  |  |  |  |  |  |  |  |  |  |  |
| 25-29 | 14.1 |  | 44.8 | 37.5 | 10.9 | 6.9 |  | 11.9 |  | 9.7 | 27.4 | 41.0 | 22.0 |
| 30-34 | 12.9 |  | 51.3 | 33.4 | 9.7 | 5.6 |  | 11.6 |  | 9.2 | 27.0 | 40.5 | 23.4 |
| 35-39 | 12.3 |  | 56.5 | 29.4 | 8.0 | 6.1 |  | 11.8 |  | 7.8 | 27.6 | 40.2 | 24.4 |
| 40-44 | 12.1 |  | 64.4 | 23.2 | 6.8 | 5.6 |  | 12.1 |  | 8.6 | 32.0 | 37.6 | 21.8 |
| 45-49 | 11.1 |  | 70.2 | 20.7 | 4.9 | 4.2 |  | 12.6 |  | 10.3 | 31.8 | 37.7 | 20.3 |
| 50-54 | 9.9 |  | 76.6 | 14.9 | 4.6 | 3.8 |  | 12.0 |  | 11.2 | 32.3 | 36.6 | 20.0 |
| 55-59 | 8.9 |  | 80.4 | 11.9 | 4.3 | 3.4 |  | 10.1 |  | 12.8 | 32.9 | 34.0 | 20.3 |
| 60-64 | 7.7 |  | 83.8 | 9.2 | 3.3 | 3.6 |  | 8.0 |  | 16.7 | 34.5 | 30.2 | 18.5 |
| 65-69 | 6.8 |  | 87.7 | 6.5 | 3.2 | 2.5 |  | 5.6 |  | 23.4 | 36.6 | 25.7 | 14.3 |
| 70-74 | 4.3 |  | 89.3 | 5.6 | 3.0 | 2.1 |  | 4.3 |  | 30.6 | 36.0 | 21.5 | 11.8 |
| Total | 100.0 |  | 66.4 | 22.2 | 6.6 | 4.8 |  | 100.0 |  | 2.1 | 31.1 | 36.2 | 20.6 |
|  |  |  |  |  |  |  |  |  |  |  |  |  |  |
| Black male | |  |  |  |  |  |  |  |  |  |  |  |  |
| 25-29 | 14.2 |  | 46.0 | 35.4 | 12.5 | 6.1 |  | 12.4 |  | 14.9 | 36.1 | 34.9 | 14.1 |
| 30-34 | 12.4 |  | 52.8 | 31.6 | 9.7 | 5.9 |  | 11.9 |  | 13.9 | 37.7 | 32.6 | 15.8 |
| 35-39 | 11.8 |  | 59.6 | 25.3 | 9.1 | 5.9 |  | 12.1 |  | 11.4 | 38.1 | 34.0 | 16.6 |
| 40-44 | 11.8 |  | 67.5 | 20.6 | 6.5 | 5.4 |  | 12.6 |  | 11.0 | 40.0 | 31.8 | 17.1 |
| 45-49 | 11.6 |  | 73.5 | 16.5 | 5.7 | 4.3 |  | 13.0 |  | 13.7 | 41.6 | 29.2 | 15.6 |
| 50-54 | 10.2 |  | 78.7 | 12.8 | 5.1 | 3.3 |  | 12.2 |  | 15.4 | 39.3 | 29.2 | 16.1 |
| 55-59 | 9.2 |  | 85.0 | 8.9 | 3.5 | 2.6 |  | 9.8 |  | 15.9 | 37.4 | 30.0 | 16.7 |
| 60-64 | 7.7 |  | 88.2 | 6.8 | 2.9 | 2.1 |  | 7.6 |  | 19.5 | 35.6 | 28.2 | 16.8 |
| 65-69 | 6.6 |  | 91.1 | 4.2 | 2.5 | 2.1 |  | 5.0 |  | 25.5 | 37.1 | 21.7 | 15.7 |
| 70-74 | 4.4 |  | 91.6 | 4.1 | 1.9 | 2.4 |  | 3.5 |  | 32.3 | 35.1 | 19.3 | 13.2 |
| Total | 100.0 |  | 69.4 | 19.4 | 6.8 | 4.4 |  | 100.0 |  | 15.4 | 38.2 | 30.5 | 15.9 |
| ^1^For each year, the first column shows the age distributions, and the rows spanning the next four | | | | | | | | | | | | | |
| columns, the education distributions within age groups. | | | | | | | | | | |  |  |  |
| ^2^less than high school | | | |  |  |  |  |  |  |  |  |  |  |
| ^3^high school degree | | | |  |  |  |  |  |  |  |  |  |  |
| ^4^some college | |  |  |  |  |  |  |  |  |  |  |  |  |
| ^5^at least four-year college | | | | |  |  |  |  |  |  |  |  |  |
