# Supplementary material for: Four Decades of Obesity Trends among Non-Hispanic Whites and Blacks in the United States: Analyzing the Influences of Educational Inequalities in Obesity and Population Improvements in Education
Source: PLoS One. 2016 Nov 28;11(11):e0167193. doi: 10.1371/journal.pone.0167193 (PMC5125692; doi:10.1371/journal.pone.0167193)
Supplement: S3 Table — (DOC) [file pone.0167193.s005.doc]

S3 Table. Actual vs. hypothetical obesity levels (95% CI), 1970 and 2010, US non-Hispanic whites and blacks, aged 25-74, 1971-2012 NHANES, 1970 Census and 2010 American Community Survey

|  |  | Whites & blacks | White female | White male | Black female | Black male |
| --- | --- | --- | --- | --- | --- | --- |
| **Year 1970** | |  |  |  |  |  |
| Actual value | | 15.2(13.9,16.5) | 15.1(13.8,16.5) | 11.8(10.1,13.4) | 30.1(26.7,33.5) | 17.6 (13.5, 21.7) |
| Standardized with 2010 age distribution | | 15.7(14.4,16.9) | 15.7(14.3,17.1) | 12.1(10.5,13.7) | 30.6(27.1,34.0) | 17.7 (13.6, 21.9) |
|  |  |  |  |  |  |  |
| **Year 2010** | |  |  |  |  |  |
| Actual value | | 38.8(37.8,39.8) | 35.4(33.8,36.9) | 37.3(34.8,39.9) | 57.5(55.1,60.0) | 40.1 (36.8, 43.3) |
| Four hypothetical scenarios | |  |  |  |  |  |
|  | 1. Using 1970 education distribution | 40.9(40.7,41.0) | 40.0(38.0,42.0) | 38.2(35.2,41.2) | 56.5(53.4,59.6) | 37.7 (34.3, 41.2) |
|  | 2. Using 1970 obesity risks | 13.1(11.6,14.5) | 10.8( 9.4,12.3) | 10.7( 8.8,12.6) | 19.6(14.5,24.7) | 20.0 (15.4, 24.6) |
|  | 3. Using 1970 educational differences in obesity^1^ | 33.6(31.3,35.8) | 28.7(25.2,32.2) | 33.9(29.9,38.0) | 59.1(50.0,68.1) | 40.1 (36.8, 43.3) |
|  | 4. Eliminating educational differences in obesity^1^ | 30.9(29.5,32.3) | 26.1(23.6,28.5) | 30.9(27.5,34.3) | 50.0(45.0,55.1) | 40.9 (36.2, 45.5) |
| ^1^Scenarios 3 and 4 used the actual values for the 2010 obese probabilities of the four-year college graduates | | | | | |  |
